# Weight a Minute: Investigating the Impact of Body Mass Index on Early Outcomes After Breast Augmentation

**DOI:** 10.1007/s00266-026-05635-3

**Published:** 2026-02-25

**Authors:** Samuel Knoedler, Jennifer A. Watson, Felix J. Klimitz, Filippo A. G. Perozzo, Thomas Schaschinger, Luzie Hoffmann, Sarah von Isenburg, Lena Schemet, Patrick Reinert, Sarah Friedrich, Omar Allam, Fortunay Diatta, Bong-Sung Kim, Martin Kauke-Navarro

**Affiliations:** 1 Department of Plastic Surgery and Hand Surgery, Klinikum Rechts der Isar, Technical University of Munich, Munich, Germany; 2 Division of Plastic Surgery, Department of Surgery, Yale School of Medicine, New Haven, CT USA; 3 Department of Plastic Surgery and Hand Surgery, University Hospital Zurich, Zurich, Switzerland; 4 Department of Hand-, Plastic and Reconstructive Surgery, Microsurgery, Burn Trauma Center, BG Trauma Center Ludwigshafen, University of Heidelberg, Ludwigshafen, Germany; 5 Department of Plastic and Reconstructive Surgery, Cleveland Clinic Foundation, Cleveland, OH USA; 6 Private Practice Isthetikum, Munich, Germany; 7 Department of Mathematical Statistics and Artificial Intelligence in Medicine, University of Augsburg, Augsburg, Germany; 8 Centre for Advanced Analytics and Predictive Sciences (CAAPS), Augsburg University, Augsburg, Germany; 9 Department of Surgery, Division of Plastic and Reconstructive Surgery, Yale New Haven Hospital, Yale School of Medicine, New Haven, USA

**Keywords:** Breast augmentation, Augmentation mammoplasty, Aesthetic breast surgery, Body mass index, BMI, NSQIP

## Abstract

**Background:**

The relationship between body mass index (BMI) and postoperative morbidity in breast augmentation remains poorly defined. This gap limits evidence-based decision-making amid rising BMI trends. Our study aims to establish a BMI-based risk threshold and quantify its impact on 30-day morbidity following aesthetic breast augmentation.

**Methods:**

We retrospectively analyzed the American College of Surgeons National Quality Improvement Program database (2009–2023). Adult female patients undergoing elective primary breast augmentation for aesthetic purposes were included. BMI cut point determination employed cubic spline modeling followed by Youden Index optimization. Propensity score matching and multivariable logistic regression were utilized to evaluate the association between BMI and 30-day postoperative outcomes.

**Results:**

Among 6,515 patients analyzed, we identified BMI ≥25.2 kg/m^2^ as a statistically derived risk threshold, with 21.0% (n=1,363) of patients exceeding this cut-point. Patients above this threshold demonstrated significantly higher baseline comorbidity burden, including hypertension (6.0% vs 2.3%, *p* < 0.001) and diabetes mellitus (2.2% vs 0.5%, *p* < 0.001). Overall 30-day morbidity was markedly elevated in the higher BMI cohort (4.3% vs 1.3%, *p* < 0.001), with corresponding increases in reoperation rates (1.9% vs 0.8%, *p* = 0.014) and unplanned readmissions (1.1% vs 0.2%, *p* < 0.001). Multivariable analysis confirmed BMI ≥ 25.2 kg/m^2^ as an independent predictor of adverse outcomes (adjusted OR 3.13, *p* < 0.001). Propensity score matching validated this association with similar effect magnitude (OR 3.35, *p* < 0.001).

**Conclusion:**

This analysis establishes BMI ≥25.2 kg/m^2^ as a clinically actionable threshold associated with a more than threefold increase in perioperative complications following aesthetic breast augmentation. These findings provide an evidence-based foundation for BMI-stratified risk assessment and informed consent protocols in breast augmentation. Implementation of enhanced perioperative surveillance and risk mitigation strategies should be considered for patients exceeding this threshold to optimize surgical outcomes and patient safety.

**Level of Evidence III:**

This journal requires that authors assign a level of evidence to each article. For a full description of these Evidence-Based Medicine ratings, please refer to the Table of Contents or the online Instructions to Authors www.springer.com/00266.

## Introduction

Breast augmentation is one of the most frequently performed aesthetic operations worldwide, with nearly 1.9 million procedures reported in 2023 [1]. Its widespread popularity highlights the need to maximize patient safety and to understand risk factors that may compromise postoperative outcomes and patient satisfaction. Among these, obesity has been consistently linked to higher rates of complications across multiple surgical disciplines, including wound dehiscence, infections, and thromboembolic events [2–6].

The mechanisms underlying this association are believed to be multifactorial. Obesity is characterized by a chronic low-grade inflammatory state, with elevated circulating cytokines such as Tumor Necrosis Factor (TNF)-α and Interleukin (IL)−6, reduced levels of protective adipokines like adiponectin, and infiltration of pro-inflammatory immune cells into adipose tissue [5, 7–9]. This inflammatory environment impairs immune and endothelial function, thereby increasing susceptibility to adverse events [10–13]. At the same time, it is also important to note that patients who have underweight tend to face an increased risk of complications, mainly due to limited physiological capacities and nutritional deficiencies [14].

Despite the plausible inference that both overweight and underweight patients may experience poorer outcomes after breast augmentation, to date, no large-scale study has explicitly investigated the perioperative significance of patient body mass index (BMI) for this procedure [15]. As a result, there are no clear guidelines regarding the safest BMI range for patients undergoing breast augmentation. This leaves surgeons uncertain about the best practices for managing patients with BMIs outside the healthy range, while patients cannot accurately anticipate the perioperative risks they may face [16]. The knowledge gap is particularly concerning in light of the increasing prevalence of obesity; by 2030, the mean BMI of surgical patients is projected to fall within the obesity class I [17]. In summary, while breast augmentation remains highly popular, and the number of surgical patients with BMIs beyond the healthy range continues to rise, the impact of BMI on outcomes after this procedure has not yet been determined.

To bridge this significant knowledge gap, our study leverages the high-volume, multi-institutional American College of Surgeons (ACS) National Surgical Quality Improvement Program (NSQIP) database. The ACS-NSQIP is a well-established research platform in the field of breast surgery and collects more than 150 data points on patients’ preoperative health, intraoperative variables, and early postoperative outcomes from hundreds of hospitals [18–23]. By utilizing this extensive dataset, we aim to investigate the correlation between BMI and outcomes following breast augmentation surgery. Although the postoperative follow-up is limited to 30 days, previous studies have demonstrated that short-term outcomes and the occurrence of adverse events in the early postoperative period significantly influence long-term patient satisfaction and well-being [24, 25]. For patients, understanding how BMI impacts surgical outcomes can lead to more informed decision-making and guidance in setting realistic expectations regarding the potential risks of breast augmentation. For surgeons, this line of research can provide valuable insights that help inform preoperative counseling, risk stratification, and surgical planning. By identifying the BMI thresholds associated with increased risks, surgeons can tailor their approach to minimize complications and optimize outcomes. Moreover, our findings can pave the way for the development of evidence-based recommendations for breast augmentation in patients with varying BMIs. Establishing clear guidelines regarding BMI and its impact on breast augmentation outcomes can help standardize care, reduce variability in surgical practices, and ultimately improve patient safety and satisfaction.

## Methods

### Data Source

The ACS-NSQIP is a high-volume, case-mix-adjusted, and outcome-based database that measures surgical success. It collects detailed and standardized clinical data on preoperative health characteristics, intraoperative variables, and 30-day postoperative outcomes from nearly 700 participating hospitals. This data is primarily used to identify areas for improvement, benchmark against national standards, and guide evidence-based practice. Trained Surgical Clinical Reviewers (SCRs) input the data directly from the medical charts of randomly selected patients. In addition, ACS-NSQIP conducts regular audits to verify the accuracy and reliability of the submitted data, identifying and correcting any discrepancies or errors to maintain high data integrity [26]. Since all data were de-identified, this study was classified as non-human subject research under the Institutional Review Board (IRB) #2000035387 (Yale School of Medicine, New Haven, CT).

### Patient Selection

We conducted a comprehensive analysis of the American College of Surgeons National Surgical Quality Improvement Program (ACS-NSQIP) database over a 15-year period (2009–2023). The study cohort was limited to adult female patients (aged ≥18 years) undergoing elective primary breast augmentation, identified by the Current Procedural Terminology (CPT) code 19325 ("Augmentation mammoplasty; with prosthetic implant") in conjunction with diagnostic codes for aesthetic breast surgery—ICD-9 codes V50.1 ("Encounter for cosmetic surgery") and 611.82 ("Breast hypoplasia"), as well as ICD-10 codes Z41.1 and N64.82.

**Fig. 1:**
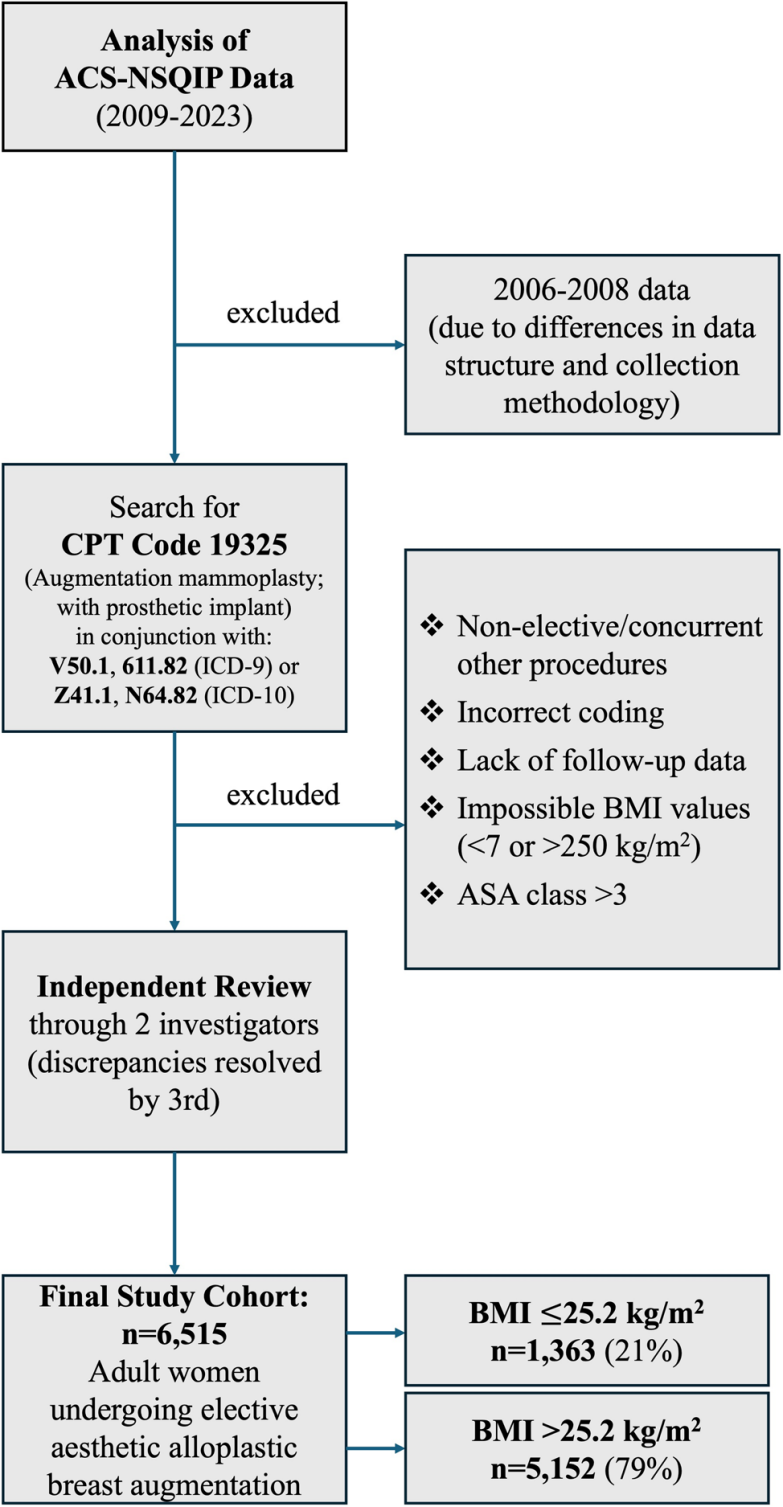
Flow diagram of patient selection, showing inclusion and exclusion criteria applied to the ACS-NSQIP dataset for identification of the final study cohort of adult women undergoing elective aesthetic breast augmentation with implants

To ensure cohort homogeneity and reduce potential confounding, stringent exclusion criteria were applied: (i) non-elective procedures; (ii) inpatient surgeries; (iii) procedures performed under non-general anesthesia; (iv) cases not performed by board-certified plastic surgeons; (v) incomplete or inaccurate procedural coding; (vi) male, non-binary, or transgender patients; (vii) missing or physiologically implausible height and/or weight data, precluding accurate body mass index (BMI) calculation; (viii) BMI values <7 or >250 kg/m^2^; (ix) patients classified as American Society of Anesthesiologists (ASA) physical status >3; (x) patients with functional dependency; and (xi) cases lacking complete 30-day postoperative follow-up data. In addition, all cases involving any concurrent invasive procedures other than breast augmentation were excluded to isolate outcomes specific to standalone augmentation procedures. Revision breast surgeries—including implant exchange, explantation, and capsular procedures—were excluded based on relevant CPT codes. Moreover, cases of breast augmentation not involving prosthetic implants, such as those performed with autologous fat grafting, were also excluded. All included cases underwent independent dual review by two investigators (S.K. and F.K.) to ensure accurate case classification. Discrepancies were resolved by a senior investigator (M.K.-N.), ensuring consistency and methodological rigor. The patient selection process is illustrated in Fig. 1.

### Variable Extraction

We collected detailed patient demographics, including age and self-identified race. Health status parameters were also extracted, encompassing a history of diabetes mellitus, chronic obstructive pulmonary disease (COPD), congestive heart failure (CHF), active dialysis, dyspnea, immunosuppressive therapy, disseminated cancer, bleeding disorders, receipt of preoperative blood transfusions (≥1 unit of whole or packed red blood cells within 72 hours before surgery), and previous sepsis. Additionally, we assessed whether patients were active smokers, evaluated their functional health status as well as ASA physical status classification. BMI was calculated using the formula [weight (pounds)/height (inches)^2^ × 703], with a BMI of ≥30 kg/m^2^ defined as obesity. The year of surgery was recorded for each case within the 15-year study period.

Operative data included the duration of surgery, and the primary outcome was the occurrence of any adverse event within 30 days postoperatively. This composite endpoint comprised reoperation, (unplanned) hospital readmission, and any surgical or medical complication. All surgical complications systematically recorded by the ACS-NSQIP were evaluated and included superficial and deep incisional surgical site infections, organ/space infections, wound dehiscence, and bleeding requiring transfusion. Similarly, all medical complications captured consistently throughout the 15-year study period were analyzed and included pneumonia, pulmonary embolism, prolonged ventilator dependence (>48 hours), acute renal failure, urinary tract infection, cerebrovascular accident/stroke, myocardial infarction, deep vein thrombosis or thrombophlebitis, sepsis, and septic shock.

### Statistical Analysis

The raw data were processed into analyzable Microsoft Excel files (V.16, Microsoft Corporation, Redmond, WA, USA) via SPSS for Windows (V.29, IBM Corporation, Armonk, NY, USA). All data were stored in an electronic laboratory notebook (LabArchives, LLC, San Marcos, CA, USA) and analyzed using the R statistical software (V. 4.1.2). Categorical data are presented as absolute numbers (n) and percentages (%), and continuous variables as mean ± standard deviation. Risk factors for any, surgical, and medical complications were evaluated exploratively using t-tests for continuous variables and chi-square tests for categorical variables.

To determine the optimal cut-off value for BMI, a two-step approach was applied. First, we modeled the influence of BMI on the risk of any complication using splines. This was performed by the R package rms to investigate whether the effect of the BMI is non-linear, and thus, two or more cut-off points might be suitable. It turns out that the risk is lowest for a BMI of 20.4 kg/m^2^. In addition, for BMI values below 18 and over 35, the uncertainty of the risk prediction increases sharply. This can be attributed to the low level of data available in these areas. In the interval between these two values, an almost linear effect can be observed, which is why the Youden-Index can be used to determine the optimal cut-off value. For this, the package cutpointr was used. The Youden-Index is ideally above 0.5, as this indicates that the test has an appropriate balance between sensitivity and specificity. To compare differences in outcomes for patients with BMI values equal to or larger than the cutpoint (25.2 kg/m^2^) versus patients with BMI values smaller than the cutpoint (25.2 kg/m^2^), we used a propensity score matching (PSM) approach. Propensity scores were calculated, adjusting for confounders (age, diabetes, hypertension, smoking, bleeding disorders, and ASA classification). Generalized full matching was performed using the package MatchIt. The closeness of the treated-untreated subjects was chosen with a caliper of 0.1 times the standard deviation of the logit of the propensity score. The causal odds ratios (ORs) for the outcomes of interest were compared for patients with BMI values equal to or larger than the cutpoint (25.2 kg/m^2^) versus patients with BMI values smaller than the cutpoint (25.2 kg/m^2^). Multivariable logistic regression, adjusting for the aforementioned confounding factors, was calculated in the matched population. Univariable, multivariable logistic regression, and sensitivity analyses were additionally calculated in the overall population. Adjusted ORs for the other risk factors were also obtained from the latter analyses. Statistical significance was set at *p* < 0.05. All reported p-values are nominal.

## Results

### Patient Demographics and Baseline Characteristics

The final cohort comprised 6,515 patients, of whom 409 patients (6.3%) were classified as underweight, 4,661 (71.5%) as normal weight, 1,121 (17.2%) as overweight, 259 (4.0%) as obesity class I, 35 (0.5%) as obesity class II, and 30 (0.5%) as obesity class III. Using the statistically derived threshold of 25.2 kg/m^2^, 5,152 patients (79.1%) were classified as having BMI ≤25.2 kg/m^2^, while 1,363 patients (20.9%) had BMI >25.2 kg/m^2^. The mean patient age was 35.1 ± 10.1 years, with statistically significant age differences across BMI categories (*p*<0.001). Patients with BMI >25.2 kg/m^2^ were older than those with BMI ≤25.2 kg/m^2^ (36.2 ± 10.5 vs 34.8 ± 10.0 years, *p*<0.001). Significant racial differences emerged across BMI categories (*p*<0.001). Black or African American patients demonstrated disproportionate representation in higher BMI groups, comprising 9.5% (n=130) of patients with BMI >25.2 kg/m^2^ compared to only 3.6% (n=184) of those with BMI ≤25.2 kg/m^2^. Conversely, Asian patients showed underrepresentation in higher BMI categories, constituting just 1.5% (n=21) of the BMI >25.2 kg/m^2^ versus 3.7% (n=192) of the BMI ≤25.2 kg/m^2^ group. Strong associations emerged between BMI categories and comorbidity prevalence. Hypertension demonstrated a clear BMI-related gradient, increasing from 2.4% in normal-weight patients to 14.2% in obesity class II patients [27]. Diabetes mellitus followed a similar pattern, progressing from 0.5% in normal-weight to 5.7% in obesity class II categories. When analyzed by the identified threshold, patients with BMI >25.2 kg/m^2^ exhibited significantly higher rates of both hypertension (6.0% vs 2.3%, *p*<0.001) and diabetes (2.2% versus 0.5%, *p*<0.001), validating the clinical relevance of this statistical threshold. Further details are shown in Table 1.

**Table 1:** Demographics and preoperative health characteristics of all patients undergoing breast augmentation

Characteristic	All patients (n=6,515)	Patients with BMI >25.2kg/m2 (n=1,363)	Patients with BMI ≤25.2kg/m2 (n=5,152)	*p*-value	Underweight (BMI <18.5 kg/m^2^) (n= 409)	Normal Weight (BMI 18.5-24.9 kg/m^2^) (n= 4,661)	Overweight (BMI 25-29.9 kg/m^2^) (n = 1,121)	Obesity class I (BMI 30-34.9 kg/m^2^) (n=259)	Obesity class II (BMI 35-39.9 kg/m^2^) (n= 35)	Obesity class III (BMI ≥40 kg/m2) (n= 30)
Demographics										
Age, mean ± SD years)	35 ± 10	36 ± 11	35 ± 10	**<0.001**	34 ± 10	35 ± 10	36 ± 10	37 ± 11	38 ± 13	37 ± 10
BMI, mean ± SD (kg/m^2^)	22.9 ± 3.8	28.5 ± 3.8	21.4 ± 2.0	**<0.001**	17.6 ± 0.8	21.7 ± 1.7	26.8 ± 1.3	31.8 ± 1.3	36.8 ± 1.1	45.9 ± 5.3
BMI										
Underweight	409 (6.3)	0 (0.0)	409 (7.9)	**<0.001**						
Normal weight	4,661 (71.5)	0 (0.0)	4,661 (90.5)							
Overweight	1,121 (17.2)	1,039 (76.2)	82 (1.6)							
Obesity I	259 (4.0)	259 (19.0)	0 (0.0)							
Obesity II	35 (0.5)	35 (2.6)	0 (0.0)							
Obesity III	30 (0.5)	30 (2.2)	0 (0.0)							
Race										
White	5,050 (77.5)	976 (71.6)	4,074 (79.1)		333 (81.4)	3,676 (78.9)	810 (72.3)	187 (72.2)	25 (71.4)	19 (63.3)
Asian	213 (3.3)	21 (1.5)	192 (3.7)		18 (4.4)	172 (3.7)	20 (1.8)	2 (0.8)	1 (2.9)	0 (0.0)
Black or African American	314 (4.8)	130 (9.5)	184 (3.6)		7 (1.7)	173 (3.7)	99 (8.8)	24 (9.3)	5 (14.3)	6 (20.0)
Native Hawaiian or Pacific Islander	7 (0.1)	2 (0.1)	5 (0.1)		0 (0.0)	5 (0.1)	2 (0.2)	0 (0.0)	0 (0.0)	0 (0.0)
American Indian or Alaska Native	14 (0.2)	7 (0.5)	7 (0.1)		0 (0.0)	7 (0.2)	6 (0.5)	1 (0.4)	0 (0.0)	0 (0.0)
Other	917 (14.1)	227 (16.7)	690 (13.4)	**<0.001**	51 (12.5)	628 (13.5)	184 (16.4)	45 (17.4)	4 (11.4)	5 (16.7)
Preoperative health and comorbiditie**s**										
Diabetes										
Orally-treated diabetes	24 (0.4)	12 (0.9)	12 (0.2)		2 (0.5)	10 (0.2)	11 (1.0)	1 (0.4)	0 (0.0)	0 (0.0)
Insulin-treated diabetes	31 (0.5)	18 (1.3)	13 (0.3)	**<0.001**	0 (0.0)	13 (0.3)	8 (0.7)	7 (2.7)	2 (5.7)	1 (3.3)
COPD	6 (0.1)	1 (0.1)	5 (0.1)	>0.99	1 (0.2)	4 (<0.1)	1 (<0.1)	0 (0.0)	0 (0.0)	0 (0.0)
CHF	1 (0.0)	0 (0.0)	1 (0.0)	>0.99	0 (0.0)	1 (<0.1)	0 (0.0)	0 (0.0)	0 (0.0)	0 (0.0)
Dialysis	0 (0.0)	0 (0.0)	0 (0.0)	>0.99	0 (0.0)	0 (0.0)	0 (0.0)	0 (0.0)	0 (0.0)	0 (0.0)
Hypertension	198 (3.0)	82 (6.0)	116 (2.3)	**<0.001**	10 (2.4)	105 (2.3)	52 (4.6)	23 (8.9)	5 (14.3)	3 (10.0)
Ascites	0 (0.0)	0 (0.0)	0 (0.0)	>0.99	0 (0.0)	0 (0.0)	0 (0.0)	0 (0.0)	0 (0.0)	0 (0.0)
Dyspnea										
With moderate exertion	14 (0.2)	4 (0.3)	10 (0.2)		3 (0.7)	7 (0.2)	2 (0.2)	2 (0.8)	0 (0.0)	0 (0.0)
At rest	302 (4.6)	81 (5.9)	221 (4.3)	**0.027**	10 (2.4)	206 (4.4)	66 (5.9)	19 (7.3)	1 (2.9)	0 (0.0)
Current smoker	747 (11.5)	157 (11.5)	590 (11.5)	**0.983**	58 (14.2)	526 (11.3)	121 (10.8)	36 (13.9)	3 (8.6)	3 (10.0)
Immunosuppressive therapy	37 (0.6)	11 (0.8)	26 (0.5)	**0.263**	1 (0.2)	24 (0.5)	8 (0.7)	4 (1.5)	0 (0.0)	0 (0.0)
Disseminated cancer	0 (0.0)	0 (0.0)	0 (0.0)	>0.99	0 (0.0)	0 (0.0)	0 (0.0)	0 (0.0)	0 (0.0)	0 (0.0)
Bleeding disorders	8 (0.1)	2 (0.1)	6 (0.1)	>0.99	0 (0.0)	6 (0.1)	2 (0.2)	0 (0.0)	0 (0.0)	0 (0.0)
Preoperative transfusions	0 (0.0)	0 (0.0)	0 (0.0)	>0.99	0 (0.0)	0 (0.0)	0 (0.0)	0 (0.0)	0 (0.0)	0 (0.0)
History of sepsis	11 (0.2)	2 (0.1)	9 (0.2)	>0.99	2 (0.5)	7 (0.2)	2 (0.2)	0 (0.0)	0 (0.0)	0 (0.0)
ASA Class										
1 – No Disturbance	3,625 (55.6)	597 (43.8)	3,028 (58.8)		232 (56.7)	2,752 (59.0)	546 (48.7)	77 (29.7)	9 (25.7)	9 (30.0)
2 – Mild Disturbance	2,756 (42.3)	709 (52.0)	2,047 (39.7)		170 (41.6)	1,840 (39.5)	547 (48.8)	162 (62.5)	21 (60.0)	16 (53.3)
3 – Severe Disturbance	134 (2.1)	57 (4.2)	77 (1.5)	**<0.001**	7 (1.7)	69 (1.5)	28 (2.5)	20 (7.7)	5 (14.3)	5 (16.7)
Functional status										
Independent	6,515 (100.0)	1,363 (100.0)	5,152 (100.0)	>0.99	409 (100)	4,661 (100)	1,121 (100)	259 (100)	35 (100)	30 (100)
Partially or totally dependent	0 (0.0)	0 (0.0)	0 (0.0)		0 (0.0)	0 (0.0)	0 (0.0)	0 (0.0)	0 (0.0)	0 (0.0)
Year of surgery										
2009	153 (2.3)	26 (1.9)	127 (2.5)		10 (2.4)	117 (2.5)	18 (1.6)	6 (2.3)	1 (2.9)	1 (3.3)
2010	110 (1.7)	14 (1.0)	96 (1.9)		7 (1.7)	88 (1.9)	11 (1.0)	3 (1.2)	1 (2.9)	0 (0.0)
2011	64 (1.0)	5 (0.4)	59 (1.1)		6 (1.5)	52 (1.1)	4 (0.4)	1 (0.4)	0 (0.0)	1 (3.3)
2012	216 (3.3)	35 (2.6)	181 (3.5)		20 (4.9)	159 (3.4)	34 (3.0)	3 (1.2)	0 (0.0)	0 (0.0)
2013	441 (6.8)	59 (4.3)	382 (7.4)		37 (9.0)	342 (7.3)	49 (4.4)	10 (3.9)	0 (0.0)	3 (10.0)
2014	523 (8.0)	81 (5.9)	442 (8.6)		42 (10.3)	397 (8.5)	68 (6.1)	11 (4.2)	5 (14.3)	0 (0.0)
2015	656 (10.1)	129 (9.5)	527 (10.2)		50 (12.2)	469 (10.1)	112 (10.0)	22 (8.5)	2 (5.7)	1 (3.3)
2016	702 (10.8)	141 (10.3)	561 (10.9)		58 (14.2)	498 (10.7)	115 (10.3)	26 (10.0)	2 (5.7)	3 (10.0)
2017	623 (9.6)	159 (11.7)	464 (9.0)		37 (9.0)	414 (8.9)	127 (11.3)	34 (13.1)	7 (20.0)	4 (13.3)
2018	690 (10.6)	147 (10.8)	543 (10.5)		35 (8.6)	497 (10.7)	121 (10.8)	26 (10.0)	3 (8.6)	8 (26.7)
2019	634 (9.7)	137 (10.1)	497 (9.6)		35 (8.6)	448 (9.6)	117 (10.4)	26 (10.0)	4 (11.4)	4 (13.3)
2020	391 (6.0)	97 (7.1)	294 (5.7)		19 (4.6)	271 (5.8)	79 (7.0)	15 (5.8)	4 (11.4)	3 (10.0)
2021	542 (8.3)	136 (10.0)	406 (7.9)		24 (5.9)	376 (8.1)	114 (10.2)	23 (8.9)	3 (8.6)	2 (6.7)
2022	468 (7.2)	116 (8.5)	352 (6.8)		19 (4.6)	327 (7.0)	86 (7.7)	34 (13.1)	2 (5.7)	0 (0.0)
2023	302 (4.6)	81 (5.9)	221 (4.3)	**<0.001**	10 (2.4)	206 (4.4)	66 (5.9)	19 (7.3)	1 (2.9)	0 (0.0)

Reported as n (%), unless otherwise stated

Statistically significant* p*-values are highlighted in bold

*SD* standard deviation, *COPD* chronic obstructive pulmonary disease, *CHF* congestive heart failure. Reported as n (%), unless otherwise stated

### Perioperative Outcomes and Complications

Patients in lower BMI categories demonstrated superior operative efficiency, with underweight and normal-weight patients requiring the shortest operative times (66 ± 30 minutes and 73 ± 42 minutes, respectively) (*p*<0.001). Patients exceeding the 25.2 kg/m^2^ threshold required significantly longer operative times compared to those below the threshold (92 ± 63 versus 73 ± 42 minutes, *p*<0.001). Overall, 124 patients (1.9%) experienced at least one 30-day complication, demonstrating clear BMI-stratified risk patterns. Complication rates increased progressively across BMI categories: from 1.2% in normal-weight, 2.8% in overweight, 9.7% in obesity class I, 2.9% in obesity class II, and 6.7% in obesity class III patients (*p*<0.001). The 25.2 kg/m^2^ threshold demonstrated strong discriminatory capacity, with patients above this cut-point experiencing significantly higher overall complication rates compared to those below (4.3% versus 1.4%, *p*<0.001). Reoperation and readmission requirements followed similar BMI-associated patterns. Likewise, surgical complications exhibited BMI-related gradients, with rates of 0.3% in normal-weight, 1.4% in overweight, 2.7% in obesity class I, 2.9% in obesity class II, and 3.3% in obesity class III (*p*<0.001). The >25.2 kg/m^2^ threshold again effectively stratified surgical complication risk, with higher BMI patients experiencing increased rates (1.8% versus 0.2%, *p*<0.001). Table 2 provides a detailed overview of the peri- and postoperative data.

**Table 2:** Operative and postoperative outcomes following breast augmentation

Characteristic	All patients (n=6,515)	Patients with BMI >25.2kg/m2 (n=1,363)	Patients with BMI ≤25.2kg/m2 (n=5,152)	*p*-value	Underweight (BMI <18.5 kg/m^2^) (n= 409)	Normal Weight (BMI 18.5-24.9 kg/m^2^) (n= 4,661)	Overweight (BMI 25-29.9 kg/m^2^) (n = 1,121)	Obesity class I (BMI 30-34.9 kg/m^2^) (n=259)	Obesity class II (BMI 35-39.9 kg/m^2^) (n= 35)	Obesity class III (BMI ≥40 kg/m2) (n= 30)
Operative time, Mean minutes ± SD	77 ± 48	92 ± 64	73 ± 42	**<0.001**	66 ± 30	73 ± 42	90 ± 63	96 ± 61	111 ± 78	112 ± 36
**Any complication**	124 (1.9)	59 (4.3)	65 (1.3)	**<0.001**	8 (2.0)	57 (1.2)	31 (2.8)	25 (9.7)	1 (2.9)	2 (6.7)
Mortality	0 (0.0)	0 (0.0)	0 (0.0)	>0.99	0 (0.0)	0 (0.0)	0 (0.0)	0 (0.0)	0 (0.0)	0 (0.0)
Reoperation	66 (1.0)	26 (1.9)	40 (0.8)	**<0.001**	5 (1.2)	35 (0.8)	10 (0.9)	15 (5.8)	0 (0.0)	1 (3.3)
Readmission	27 (0.4)	16 (1.2)	11 (0.2)	**<0.001**	2 (0.5)	9 (0.2)	8 (0.7)	8 (3.1)	0 (0.0)	0 (0.0)
Unplanned readmission	26 (0.4)	15 (1.1)	11 (0.2)	**<0.001**	2 (0.5)	9 (0.2)	7 (0.6)	8 (3.1)	0 (0.0)	0 (0.0)
**Surgical complication**	37 (0.6)	25 (1.8)	12 (0.2)	**<0.001**	0 (0.0)	12 (0.3)	16 (1.4)	7 (2.7)	1 (2.9)	1 (3.3)
Superficial incisional infection	20 (0.3)	14 (1.0)	6 (0.1)	**<0.001**	0 (0.0)	6 (0.1)	11 (1.0)	2 (0.8)	0 (0.0)	1 (3.3)
Deep incisional infection	3 (0.0)	2 (0.1)	1 (0.0)	0.216	0 (0.0)	1 (<0.1)	0 (0.0)	2 (0.8)	0 (0.0)	0 (0.0)
Organ space infection	4 (0.1)	3 (0.2)	1 (0.0)	0.041	0 (0.0)	1 (<0.1)	2 (0.2)	1 (0.4)	0 (0.0)	0 (0.0)
Dehiscence	7 (0.1)	6 (0.4)	1 (0.0)	**<0.001**	0 (0.0)	1 (<0.1)	3 (0.3)	2 (0.8)	1 (2.9)	0 (0.0)
Bleeding/Blood transfusion	1 (0.0)	0 (0.0)	1 (0.0)	>0.99	0 (0.0)	1 (<0.1)	0 (0.0)	0 (0.0)	0 (0.0)	0 (0.0)
**Medical complication**	22 (0.4)	10 (0.8)	12 (0.2)	**0.012**	2 (0.5)	10 (0.2)	4 (0.4)	6 (2.4)	0 (0.0)	0 (0.0)
Pneumonia	2 (0.0)	2 (0.1)	0 (0.0)	0.060	0 (0.0)	0 (0.0)	1 (<0.1)	1 (0.4)	0 (0.0)	0 (0.0)
Pulmonary embolism	3 (0.0)	1 (0.1)	2 (0.0)	>0.99	0 (0.0)	2 (<0.1)	1 (<0.1)	0 (0.0)	0 (0.0)	0 (0.0)
Ventilator dependence >48 hours	0 (0.0)	0 (0.0)	0 (0.0)	>0.99	0 (0.0)	0 (0.0)	0 (0.0)	0 (0.0)	0 (0.0)	0 (0.0)
Renal failure	1 (0.0)	0 (0.0)	1 (0.0)	>0.99	1 (0.2)	0 (0.0)	0 (0.0)	0 (0.0)	0 (0.0)	0 (0.0)
Urinary tract infection	14 (0.2)	5 (0.4)	9 (0.2)	0.301	1 (0.2)	8 (0.2)	2 (0.2)	3 (1.2)	0 (0.0)	0 (0.0)
Cerebral vascular accident/stroke	0 (0.0)	0 (0.0)	0 (0.0)	>0.99	0 (0.0)	0 (0.0)	0 (0.0)	0 (0.0)	0 (0.0)	0 (0.0)
Myocardial infarction	0 (0.0)	0 (0.0)	0 (0.0)	>0.99	0 (0.0)	0 (0.0)	0 (0.0)	0 (0.0)	0 (0.0)	0 (0.0)
DVT/Thrombophlebitis	1 (0.0)	1 (0.1)	0 (0.0)	0.475	0 (0.0)	0 (0.0)	0 (0.0)	1 (0.4)	0 (0.0)	0 (0.0)
Sepsis	2 (0.0)	2 (0.1)	0 (0.0)	0.060	0 (0.0)	0 (0.0)	1 (<0.1)	1 (0.4)	0 (0.0)	0 (0.0)
Septic shock	0 (0.0)	0 (0.0)	0 (0.0)	>0.99	0 (0.0)	0 (0.0)	0 (0.0)	0 (0.0)	0 (0.0)	0 (0.0)

Statistically significant *p*-values are highlighted in bold

Reported as n (%), unless otherwise stated

*SD* standard deviation

### Multivariable Risk Analysis and Propensity Score Matching

**Fig. 2:**
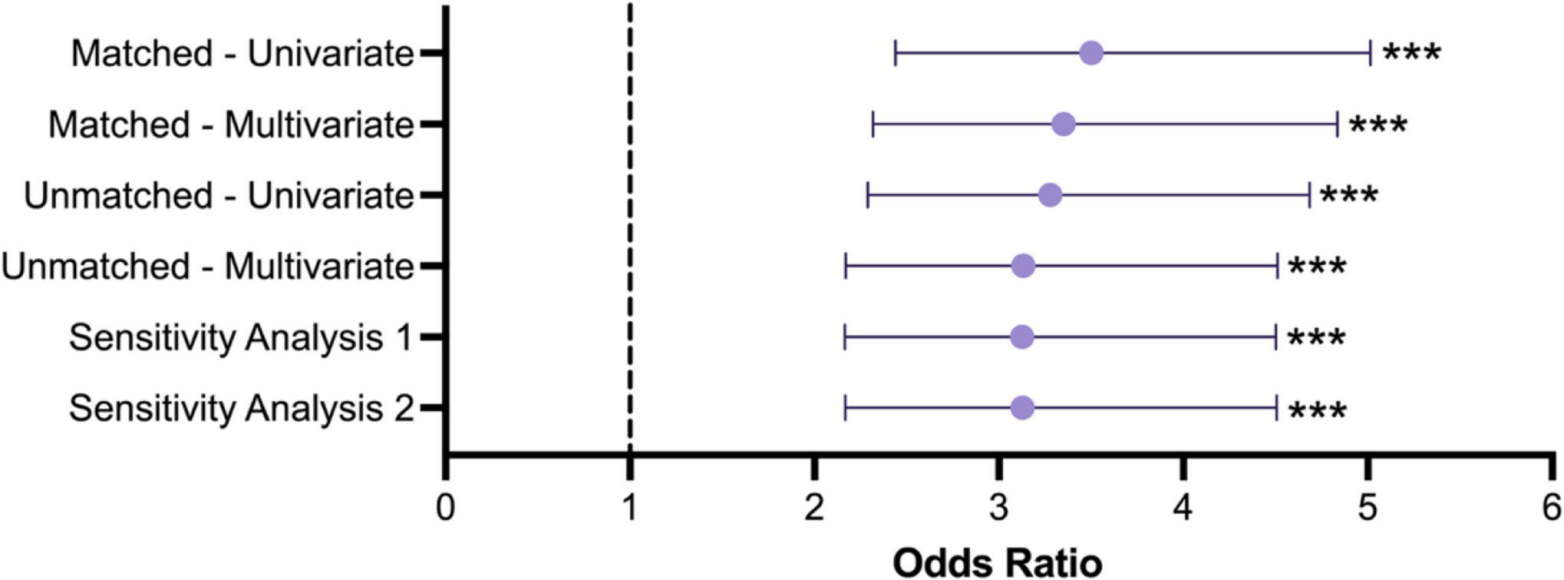
Odds ratios (ORs) with 95% confidence intervals (CIs) for complications in patients with BMI ≥ 25.2 kg/m^2^ compared to those with BMI < 25.2 kg/m^2^ (reference group). Results are shown for univariate and multivariate models in both matched and unmatched cohorts, as well as two sensitivity analyses. All data are presented in Table 3. ***p < 0.001

Our analysis identified a BMI of 25.2 kg/m^2^ as a statistically derived threshold for stratifying perioperative risk in breast augmentation. The risk of complications was found to be lowest at a BMI of 20.4 kg/m^2^. Multivariable logistic regression of the complete, unmatched dataset, adjusted for demographic variables, comorbidities, and operative characteristics, confirmed that BMI ≥25.2 kg/m^2^ is a significant independent predictor of 30-day postoperative complications. Patients exceeding this threshold demonstrated a 3.13-fold increase in the odds of experiencing any complication compared to those below it (adjusted OR 3.13, 95% CI 2.17–4.51, *p*<0.001). In addition, propensity score matching was performed, successfully balancing baseline characteristics between BMI groups. In the propensity-matched cohort, BMI ≥25.2 kg/m^2^ remained a highly significant predictor of complications, with a consistent and robust effect magnitude (adjusted OR 3.35, 95% CI 2.32–4.84, *p*<0.001). Further sensitivity analyses yielded consistent and robust findings, confirming the reliability of the primary results. All results are presented in Table 3 and Fig. 2.

**Table 3:** Odds ratios (OR) with 95% confidence intervals (CI) and *p*-values for complications in patients with BMI ≥ 25.2 kg/m^2^ vs. < 25.2 kg/m^2^, analyzed using univariate and multivariate models (both matched and unmatched), including sensitivity analyses. Sensitivity Analysis 1 and 2 represent additional multivariate analyses of the unmatched cohort, each adjusting for different sets of confounders. The BMI < 25.2 kg/m^2^ group serves as the reference for comparison in each analysis. All *p*-values were < 0.001 across analyses

Analysis type	Odds ratio (OR)	95% CI (Lower–Upper)	*p*-Value
Matched–Univariate	3.501	2.439–5.013	< 0.001
Matched–Multivariate	3.352	2.317–4.835	< 0.001
Unmatched–Univariate	3.278	2.289–4.686	< 0.001
Unmatched–Multivariate	3.132	2.169–4.511	< 0.001
Sensitivity Analysis 1	3.126	2.166–4.502	< 0.001
Sensitivity Analysis 2	3.128	2.167–4.505	< 0.001

## Discussion

According to statistics from the International Society of Aesthetic Plastic Surgery (ISAPS), the number of breast augmentation procedures performed annually grew by 50% since 2010, from 1,262,779 cases in 2010 to 1,892,777 in 2023 [1]. With the increasing demand for this procedure, the imperative to optimize its safety becomes ever more important. One well-established factor influencing postoperative outcomes is the patient’s BMI. However, while the correlation between higher BMI and increased complication rates has been thoroughly documented across a plethora of surgeries [2–6], the association between BMI and outcomes following breast augmentation has not yet been adequately explored [16]. This knowledge gap is particularly concerning given the increasing demand for breast augmentation and the rising average BMI in the surgical patient population [28]. In light of these dynamics, we sought to investigate the link between patient BMI and complication occurrence after breast augmentation surgery in more than 6,500 cases from the well-established multi-institutional NSQIP [29, 30].

It is important to acknowledge that, in general, fewer patients with elevated BMI undergo breast augmentation [6, 31, 32]. This trend is likely explained by the fact that breast augmentation is predominantly sought by women with smaller breasts, which is known to correlate with BMI [33]. Consequently, patients with higher BMIs are less likely to seek breast augmentation, which may explain why the existing literature on the association between BMI and breast augmentation outcomes is relatively sparse, as surgeons encounter patients with BMIs outside the healthy threshold less frequently in this specific context. This is corroborated by our study, in which the mean BMI of patients was 22.9 kg/m^2^, falling within the healthy normal range. However, as the surgical population’s average BMI continues to rise [34], it is expected that more and more patients with higher BMIs will seek breast augmentation. Therefore, the findings of this study will become increasingly relevant, ensuring that surgeons are equipped with the necessary knowledge to safely perform breast augmentation in patients with varying BMIs, while enabling patients to make informed decisions.

In the multifaceted field of breast surgery, obesity has been identified as a significant risk factor for complications across a wide range of procedures, including mastectomies [35, 36], implant-based and autologous breast reconstruction, as well as breast reduction [6, 37–39]. However, breast augmentation remained understudied in this regard, likely due to the reasons outlined above. Nonetheless, some studies examined the association between BMI and breast augmentation outcomes, primarily as part of broader risk assessments [40, 41].

Gupta et al. queried the insurance claims-based CosmetAssure database between 2008 and 2013 to investigate the safety of aesthetic surgery in overweight patients [42]. In their study, the majority of patients undergoing breast augmentation had a healthy BMI, yet nearly one-fourth had a BMI ≥25 kg/m^2^. This group of patients, classified as having overweight or obesity, exhibited a slightly higher complication rate of 1.6% compared to 1.4% reported for those with a BMI <25 kg/m^2^. Similarly, Hanemann et al. [43] Grotting also leveraged the CosmetAssure database (2008-2009) to examine the risk profile of breast augmentation, dichotomizing the patient cohort into those with a BMI above and below 30 kg/m^2^. The complication rate for patients without obesity was 1.9%, whereas those with obesity had a complication rate that was 2.2 times higher, at 4.2%. The difference was even more pronounced when looking specifically at postoperative infection rates, whereby the risk for patients with obesity was nearly five times higher than for those with a normal BMI (1.9% versus 0.4%). Similarly, Montemurro et Pietruski shared their 12-year experience of breast augmentation surgery in 1,212 patients. Their descriptive statistics revealed that patients with a BMI <25 kg/m^2^ faced half the risk of complications compared to those with overweight and obesity (6.8% vs. 13.3%) [44]. However, despite these marked differences, the authors were unable to establish higher BMI values as independent risk factors through multivariate logistic regression analysis. Valente et al., in their retrospective cohort analysis of the Tracking Outcomes and Operations for Plastic Surgeons (TOPS) database, identified obesity as a significant and independent risk factor for complications after breast augmentation [45]. More specifically, women with obesity were twice as likely to experience complications compared to those with normal or underweight BMI. Another study from Brazil analyzed risk factors for the explanation of breast implants in 138 patients who had undergone aesthetic breast augmentation, noting that patients with overweight or obesity were 1.5 times more likely to have their implants removed [46].

Our study contributes to the growing body of evidence establishing a robust correlation between elevated BMI and adverse postoperative outcomes of breast augmentation. Importantly, we are the first to propose a statistically derived BMI cut-off value—specifically, 25.2 kg/m^2^—above which the risk of complications increases significantly. This threshold was identified through rigorous statistical modeling and validated in both multivariable and propensity score–matched analyses. Notably, our analysis also revealed that the lowest complication risk was observed at a BMI of 20.4 kg/m^2^, further reinforcing the nonlinear and threshold-dependent nature of the BMI–risk relationship. Furthermore, among the 110 patients in our cohort who experienced postoperative complications, more than half had a BMI outside the healthy range (i.e., BMI <18.5 or ≥25 kg/m^2^), highlighting the disproportionate burden of adverse outcomes among individuals at the extremes of the BMI spectrum.

These findings carry important clinical implications for both patients and surgeons. The strong and quantifiable association between BMI and postoperative complication risk, paired with the identification of a discrete BMI inflection point, can serve as a valuable tool in preoperative risk stratification. Surgeons may need to exercise heightened vigilance when evaluating candidates for breast augmentation who fall above this threshold, particularly those classified as obese. In such cases, more comprehensive preoperative assessments, including metabolic evaluation and optimization of modifiable risk factors, may be warranted. Moreover, our results underscore the need for integrating BMI as a core variable in shared decision-making discussions, allowing patients to make more informed choices regarding their surgical care. Beyond individual patient management, these data may contribute to the refinement of evidence-based clinical guidelines for breast augmentation. As our understanding of BMI-related surgical risk continues to evolve, future studies should aim to externally validate these findings across diverse populations and surgical techniques. Such research will be essential for developing nuanced, BMI-sensitive recommendations that optimize safety and outcomes for patients across the entire BMI spectrum.

## Limitations

When interpreting and contextualizing the herein-presented results, the following limitations ought to be considered. First, the reliance on the NSQIP database with standardized data collection, which, while comprehensive, may not capture all relevant details specific to breast augmentation, such as implant type, surgical technique, including incision type and implant position, or surgeon experience, all of which could influence outcomes. Second, the database primarily includes data from U.S. institutions, potentially limiting the generalizability of our findings to other populations with different healthcare systems. Third, the relatively small number of patients at the extremes of the BMI spectrum, particularly those classified as underweight and those within obesity classes II and III, limits the statistical power to detect subtle yet potentially meaningful differences in complication rates. Nevertheless, we believe that including these subgroups contributes to a more complete and nuanced representation of postoperative outcomes across the entire BMI continuum and may encourage future studies to further explore these underexamined patient populations. Fourth, although we observed a significant association between BMI and postoperative complications, the observational design of the study limits the ability to infer causality. Furthermore, the dichotomized BMI threshold was derived from a relatively low Youden index (0.29), indicating limited discriminatory power. Therefore, the validity of this exact threshold should be interpreted with caution, and the potential influence of other confounding factors cannot be excluded. Fifth, patient-reported outcomes, which are crucial in aesthetic surgery, were not available in the database, limiting our ability to assess patient satisfaction or quality of life following the procedure. Finally, the follow-up in the NSQIP is limited to the first postoperative month, leaving any long-term complications (i.e., >30 postoperative days) unrecognized [47].

## Conclusion

Our study demonstrates a clear and statistically significant association between higher BMI and an increased risk of complications following breast augmentation surgery. Through comprehensive analysis, we identified a specific BMI threshold of 25.2 kg/m^2^, beyond which the likelihood of postoperative complications rises markedly. This finding not only provides a quantifiable risk stratification tool but also highlights the clinical relevance of BMI as an independent predictor of surgical outcomes.

## Data Availability

The datasets analyzed during this study are available from the corresponding author upon reasonable request. The ACS-NSQIP database is available through the American College of Surgeons National Surgical Quality Improvement Program with appropriate institutional participation and data use agreements.

## Abbreviations

ACS-NSQIP: American college of surgeons national surgical quality improvement program
ASA: American society of anesthesiologists
BMI: Body mass index
CHF: Congestive heart failure
CI: Confidence interval
COPD: Chronic obstructive pulmonary disease
CPT: Current procedural terminology
DVT: Deep vein thrombosis
ICD: International classification of diseases
IRB: Institutional review board
ISAPS: International society of aesthetic plastic surgeons
OR: Odds ratio
PSM: Propensity score matching
SD: Standard deviation
TOPS: Tracking outcomes and operations for plastic surgeons
